# Low Elevation Angle Estimation with Range Super-Resolution in Wideband Radar

**DOI:** 10.3390/s20113104

**Published:** 2020-05-31

**Authors:** Sha Huan, Man Zhang, Gane Dai, Huaguo Gan

**Affiliations:** 1 School of Physics and Electronic Engineering, Guangzhou University, Guangzhou 510006, China; speeshuan@gzhu.edu.cn (S.H.); 2111819028@e.gzhu.edu.cn (H.G.); 2 School of Electronics and Communication Engineering, Sun Yat-sen University, Guangzhou 510006, China; daige3@mail2.sysu.edu.cn

**Keywords:** low elevation, angle estimation, wideband radar, super-resolution

## Abstract

Height detection of a low elevation angle target is crucial in radar applications. Due to the presence of the multiple path reflections, elevation angle estimation is difficult with conventional narrowband radar waveforms. The reflection ground area parameters are especially hard to obtain for modeling. In this paper, we proposed a wideband, low elevation angle estimator based on range super-resolution, achieving a high robustness to variations in reflection coefficients. A relaxation (RELAX) algorithm was applied as the range super-resolution algorithm to separate the direct target echo and the reflected signal thanks to the relatively abundant frequency diversity. The grazing angle was obtained by synthesizing the steering vector of the direct signal and the range structure relationship between the two signal components. Theoretical analysis and simulation results confirmed the improved behavior of the proposed robust estimator in contrast to other conventional algorithms.

## 1. Introduction

Low elevation radar tracking has become an urgent and important issue since the 1970s [1,2]. A considerable amount of research has been devoted to this complex multipath problem [3,4,5]. The radar signal travels from target to radar in direct and indirect paths. The indirect paths consist of specular and diffuse components, where the former dominates in the case of a low grazing angle. It is difficult to perform angle estimation when the elevation of the target is so low that the angular separation between the direct signal and the specular reflection signal is less than 0.8 of an antenna beam width [6]. Here, the conventional mono-pulse radar fails to perform tracking due to its weak capacity to cope with such closely spaced and correlated waveforms [7].

Many methodologies have been developed for tracking low elevation targets based on array signal processing, and are mainly classified as subspace-based methods and maximum likelihood (ML) estimators. Subspace-based methods are also called non-parametric methods, such as multiple signal classification algorithm(MUSIC) and estimation of signal parameters via rotational invariant techniques (ESPRIT) [8]. Generally, use of these methods can yield an angle resolution of less than one beam-width. However, the snapshots are usually inadequate for a full rank covariance matrix in radar applications. Meanwhile, the signal coherence may cause the covariance matrix to ill-condition. A spatial smoothing technique can be used to mitigate this problem [9]. Unfortunately, this technique degrades the array gain and resolution and introduces biases to the estimates. On the other hand, a parametric model takes the height of the antenna, reflection coefficients and so on as a priori information, which works with a few snapshots and correlated sources. A refined maximum likelihood (RML) technique has been proposed based on a refined model [10]. This model combines the geometric information and physical parameters, such as the refractivity gradient, the reflection coefficient, and the divergence factor [11]. Using this a priori information contributes to good performance. However, in practice, the environmental parameters are difficult or even impossible to obtain. Vertical polarization, however, is usually not recommended as an alternative in low-angle estimation for its relatively fluctuating reflection coefficient both in amplitude and phase compared to horizontal polarization [12]. Differences in surface coverage also perturb of the steering vector of the reflected wave. The uncertainty of the model leads to estimator failure, which is highly undesirable. Many approaches, including carrying out multiple iterations and approximations, have been proposed to solve this problem. Sparse Bayesian learning has been used to estimate the perturbation and elevation angle iteratively [13,14]. A robust maximum likelihood estimator involving the use of the minimax approach has been developed [15]. Alternatively, the projection gradient method has been used to estimate jointly the reflecting surface height and target height [16]. Two approximate maximum likelihood algorithms have been proposed for a joint estimation of the angle and Doppler in partially known additive noise [17].

The methodologies mentioned above have improved the quality of the estimations to various extents. However, most of them suffer from being sensitive to the reflection coefficient, which needs to be estimated in advance. Moreover, there are not many works on broadband low elevation angle estimation. To achieve a suppression of grating lobes in low angle estimation, a noncoherent synthesis method is extracted from multiple frequency points [18]. A frequency-agile algorithm using the RML model and involving adaptively adjusting the operating frequency during target tracking has also been developed. It can minimize the mean squared errors (MSEs) of the angle estimator [19]. However, few of these works fully utilize the target information in the wideband multi-frequency signals.

In frequency diversity radar systems, the operating frequency is agile in a relatively wide frequency band, bringing about good detection and countermeasures performance. For wideband angle detection, the incoherent signal subspace method (ISSM) uses narrowband techniques on each frequency point, and finally combines the results incoherently [20]. It is not an optimal solution since full usage of the coherent signals is not achieved. The coherent signal subspace method (CSSM) [21] introduces a focusing step before estimating the covariance matrix at every frequency bin. Decoherence of the signal is achieved during focusing. Then, MUSIC is used on the entire focused narrowband covariance matrix. Both rotational signal subspace (RSS) [22] and two-sided correlation transformation (TCT) [23] are based on this focusing method, but they use two different ways to construct the focusing matrix. Several preliminary angle estimates of the target are needed to form the steering matrix for the focus.

The difference between the range of the direct wave and that of the reflected wave is determined geometrically from the target angle and the radar height, with this difference being small due to the low elevation angle. Since the use of wideband radar can provide a higher range resolution, a super-resolution algorithm was introduced here to further improve the range resolution. In this case, it became feasible to extract the angle information from the range difference. Many super-resolution methods have been used for image formation [24], and with some of them, like Capon and the amplitude and phase estimator (APES) [25,26], the resolution levels are not significantly improved due to their nonparametric nature. RELAX [27,28,29] was chosen in the current work as the super-resolution algorithm with an extended range resolution, which can not only recover the amplitude and phase of the signal in each element, but also effectively improve the performance for range estimation as an iterative optimization algorithm similar to the greedy algorithm.

In order to use wideband radar to track a low elevation angle target, we proposed using an angle estimation algorithm based on range super-resolution. The RELAX algorithm was used to further improve the range resolution for low elevation angle tracking. The energy of the direct wave and that of the reflected wave are separated in the distance dimension. Focus processing was performed on each of the two waves respectively. The algorithm obtained the angle values by synthesizing the steering vector of the direct wave and the range structure relationship between the two waves without any reflection parameter estimation. Thus, this algorithm was found to be more robust to various ground covers and more flexible for polarization waveform applications.

The rest of the paper is organized as follows. In Section 2, the multipath propagation model is built. In Section 3, the derivation of the multi-frequency refined maximum likelihood principle is described. Based on the sensitivity of the reflection coefficient, a robust angle estimator is proposed based on the range super-resolution for wideband radar systems. In Section 4, we analyze the parameter selection and the performance of the algorithm, and in Section 5, simulation results are presented to validate the proposed method.

## 2. Multipath Propagation Model

**Figure 1:**
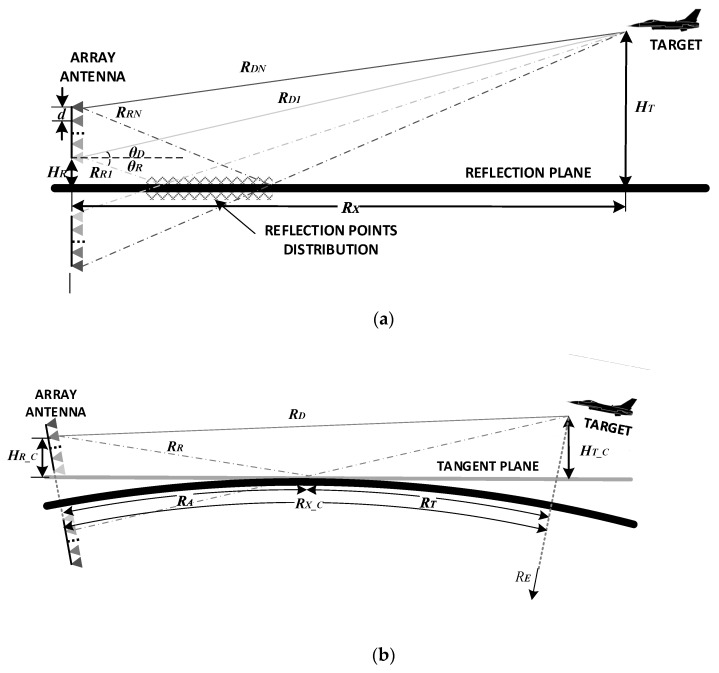
The geometry of the multipath propagation model. (**a**) Flat-Earth model; (**b**) Curved-Earth model.

First, the multipath propagation models are discussed. The propagation of the electromagnetic wave between radar and target is two-way, and this means that four distinct paths occur from the transmitter antenna to the target and back to the array element. These four paths are composed of the transmitting multipath and the receiving multipath. For the phased array radar system, the impact of the transmitting multipath could be reduced by upturning the transmitting beam. The model in this paper only considers the one-way beacon propagation model [1,2] from the target to the radar to simplify analysis. Since the mechanisms of transmitting and receiving multipath are similar, the model and the algorithm proposed in this paper can be extended to the two-way propagation scenario. The geometries for the flat-Earth model and the curved-Earth model are illustrated in Figure 1. The propagation medium was assumed to be linear, homogeneous, and isotropic within a wide frequency band. Meanwhile, the target location is at a great distance from the receiving antenna so that the direct and reflected signals can be considered as plane waves.

The reflected signals consist of the specular and the diffuse components. The specular component is highly correlated with the direct wave and is usually referred to as the coherent part of the reflected signal. The diffuse energy scattered from the irregular surface at random angles, which is the incoherent part of the reflected signal. When the reflection area is relatively smooth, like an ocean or lake surface with negligible wave disturbance, flat desert surfaces, and snow-covered terrains, the specular component dominates. Although the diffuse reflection is omitted in this paper, it will be added to the model in the next stage of research for complex terrain.

In the flat-Earth model shown in Figure 1a, the echo of the target would reach each element along two paths. The reflection points of different array elements were distributed on the reflection plane, which is grid marked. Here, it is assumed that there is only one reflected path from the target for each array element. The diffuse component was treated as white Gaussian noise for simplicity. The width of the area was related to the array size and the target angle. For the relatively low grazing angles, the reflection point distribution area would be large. Such a result causes differences between the reflection coefficients corresponding to each element.

A uniform linear array (ULA) was applied in the current work, as shown in Figure 1, which could be generalized to non-ULA models as well. Based on the assumptions above, in the flat-Earth model, the distance along the direct path between the *n*th array element and the target may be calculated using the equation

(1) $$R_{Dn}=R_{D1}-\left(n-1\right)dsin\theta_{D}$$

where $R_{D1}$ is the target range information obtained using acquisition mode, and the array consists of *N* sensors, *d* is the distance between the two array elements, equaling to a half wavelength of the highest frequency in the bandwidth. *θ_D_* is the angle of incidence of the direct signal. The distance along the reflected path between the nth array element and the target is

(2) $$R_{Rn}=R_{R1}-\left(n-1\right)dsin\theta_{R}$$

where *θ_R_* is the incident angle of the reflected signal. Generally, the target is tens or hundreds of kilometers away, and the height of the radar is much smaller than that of the target. The approximation

(3) $$\theta_{D}\approx -\theta_{R}$$

can be made. The difference between these two paths was then derived, as shown in Appendix A, to be

(4) $$\Delta R_{n}\approx 2sin\theta_{D}[H_{R}+\left(n-1\right)d]$$

According to Equation (4), the path difference was determined from the array elements heights and the grazing angle.

In the curved-Earth model, the Earth can be replaced by the tangent plane of the reflection point. The difference between the two paths turns out to be:

(5) $$\Delta R_{n\_C}\approx 2\frac{H_{T\_C}}{R_{D}cos\theta_{D}}[H_{R\_C}+\left(n-1\right)d]\approx 2\frac{\left(H_{T}-\frac{R_{T}^{2}}{2R_{E}}\right)}{R_{D}cos\theta_{D}}[H_{R}+\left(n-1\right)d-\frac{R_{A}^{2}}{2R_{E}}]$$

where *R_A_* and *R_T_* represent the distances along the curve from the reflection point to the array and the target, respectively. They can be calculated iteratively by using Newton’s method [18] to solve a cubic equation. *R_E_* is the imaginary Earth radius, which can be calculated from the real Earth radius and the refractivity gradient, and $H_{T\_C}$ is the virtual height of the target relative to the tangent plane. The actual height can be calculated by Equation (5).

The discussion below was developed based on the flat-Earth model for simplicity.

## 3. Wideband Multifrequency Low-Angle Estimation

### 3.1. Multifrequency Signal Equation

Assume that the entire bandwidth is *B* and the frequency spectrum interval is Δ*f*. The wideband waveform consists of *M* frequency points uniformly distributed in the whole band, giving the equation (M−1)Δf=B. It is essential that the subsequent angle estimation starts only after a target is detected. Moreover, due to the target being at low altitudes, the effects of the antenna pattern are not taken into account for the small grazing angle compared with the beam width. Meanwhile, the propagation losses experienced by the direct and reflected waves are assumed to be identical for the small range difference between them. Under these assumptions, the signal received by the *n*th element at the *m*th frequency point can be written as

(6) $$S_{mn}=Aexp\left\{-j2\pi \frac{f_{m}}{c}R_{Dn}\right\}+A\rho exp\left\{-j2\pi \frac{f_{m}}{c}R_{Rn}\right\}+n_{mn}$$

where A denotes the complex amplitude of the received signal from the direct path, *ρ* is the complex reflection coefficient assumed to be same for all the reflected rays in a simplified model, *f_m_* represents the *m*th frequency point, *c* is the light speed, and *n_mn_* is the zero-mean white complex Gaussian noise. Furthermore, the noises for different frequencies and elements are assumed to be independent.

Using the vector/matrix form expressions

$$f={\left[\begin{array}{c} f_{1}\\ \begin{array}{c} .\\ .\\ f_{m} \end{array}\\ \begin{array}{c} .\\ .\\ f_{M} \end{array} \end{array}\right]}^{T}\in {\mathbb{C}}^{1\times M}d=\left[\begin{array}{c} 0\\ \begin{array}{c} .\\ .\\ \left(n-1\right)d \end{array}\\ \begin{array}{c} .\\ .\\ \left(N-1\right)d \end{array} \end{array}\right]\in {\mathbb{C}}^{N\times 1}N=\left[\begin{array}{ccc} n_{11} & \cdots & n_{M1}\\ \vdots & \ddots & \vdots \\ n_{1N} & \cdots & n_{MN} \end{array}\right]\in {\mathbb{C}}^{N\times M}$$

**Figure 2:**
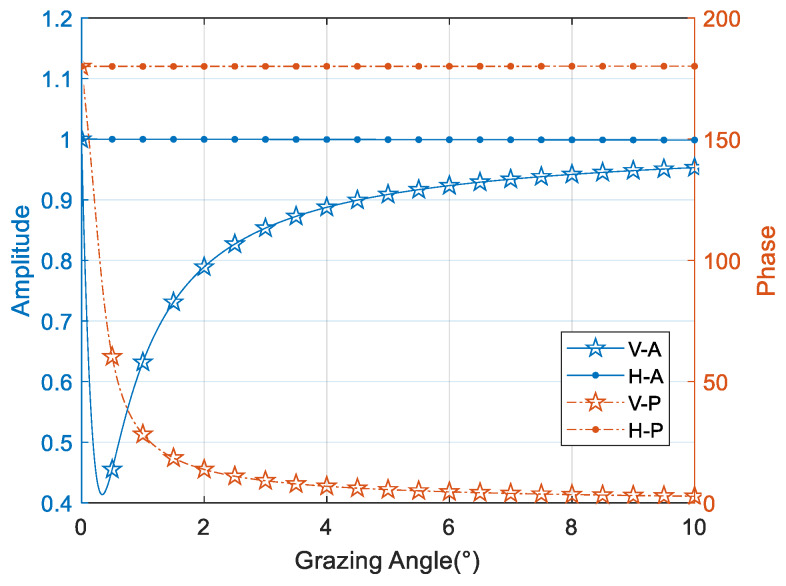
Reflection coefficients properties versus grazing angle at 9 GHz.

In the following, the grazing angle is abbreviated as *θ*. Then, Equation (6) can be rewritten in a *N* × *M* matrix form below

(7) $$S=Aexp\left\{-j2\pi \left(R_{D1}-dsin\theta \right)\frac{f}{C}\right\}+A\rho exp\left\{-j2\pi \left(R_{D1}+dsin\theta +2H_{R}sin\theta \right)\frac{f}{C}\right\}+N$$

where $H_{R}$ is the radar height, while *ρ* is the reflection coefficient determined mainly from the Fresnel reflection coefficient along with the surface roughness factor and the divergence factor [18]. Without considering roughness and divergence impact, *ρ* in the X-band can be obtained by analytical calculation [12]. Significantly different properties in the horizontal and vertical polarizations are shown in Figure 2. Unlike horizontal polarization (marked with dots), which is shown not depend much on grazing angle, the vertical polarization (marked with stars) changes significantly with changing grazing angle, especially between 0 and 4 degrees. So, most researchers prefer using horizontal polarization in low-angle estimation because of its relatively stable characteristics both in amplitude and phase, but it also limits the application of the corresponding algorithms.

**Figure 3:**
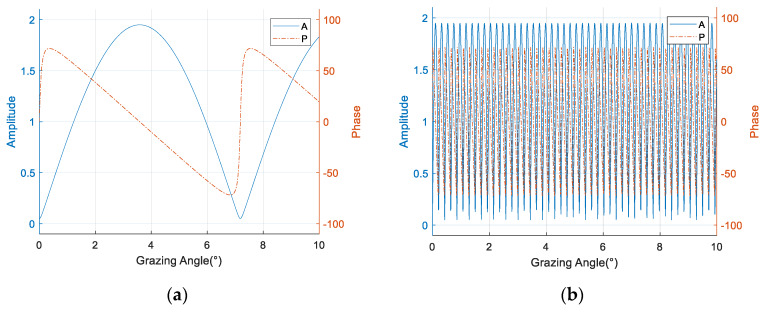
The impact of the modulation term in the different working frequency. (**a**) 300 MHz; (**b**) 9 GHz.

The modulation term of the common steering vector in (7) can be expressed as

(8) $$\begin{array}{c} 𝓂\left(\theta \right)=1+\rho \exp\left\{\frac{-j4\pi sin\theta \left(H_{R}+d\right)f}{C}\right\}\\ =1+\rho -2\rho sin\left(\frac{2\pi sin\theta (H_{R}+d)f}{C}\right)e^{j\left(\frac{\pi }{2}-\frac{2\pi sin\theta (H_{R}+d)f}{C}\right)} \end{array}$$

where 𝓂(θ) is used to introduce a periodic modulation effect on the direct path signal, which leads to a phase flip along the angle axis. The frequency of the periodic modulation consists of two factors, namely the working frequency and the array element height, and thus the phase inversions of different array elements, are not synchronized. The amplitude and phase of *ρ* determine the modulation depth and additive phase respectively. Figure 3 demonstrates the effects of the modulation term on the single array element at 300 MHz and 9 GHz. The modulation becomes obviously denser as the working frequency is increased, and the resulting angular ambiguity will invalidate the phase division-based beam splitting algorithm [6] at high frequencies.

### 3.2. Refined ML Estimator

The refined ML estimator (RML) was used to determine target height by finding the maximum correlation between the received array signals and the simulated signatures based on the highly refined module in Section 3.1. This method constructs a noise-free signal $u_{m}\left(\theta \right)$ based on the model above, incorporating specific propagation parameters, namely radar height, target range, the reflection coefficient *ρ*, and the characteristics of the radar. The ML estimate of the angle is the one corresponding to the largest peak of the following equation. The maximum likelihood results between different frequency points are directly accumulated, forming the basis of the final angle estimation [18].

(9) $$\hat{\theta }=\underset{\theta }{\arg\;\max}\frac{1}{\sum_{m=1}^{M}\frac{\|s_{m}{\|}_{2}}{\sigma_{m}^{2}}}\overset{M}{\underset{m=1}{\sum }}\frac{\left\|s_{m}^{H}u_{m}{\left(\theta \right)}_{2}\right\|}{\sigma_{m}^{2}\|u_{m}{\left(\theta \right)\|}_{2}}$$

where ${\left\|\cdot \right\|}_{2}$ denotes the Euclidean norm, which is the square root of the sum of squares.

Among all these parameters, *ρ* is most closely related to the estimator performance, however, it is difficult to be estimated accurately with a small number of snapshots. In actual application scenarios, the reflection coefficients according to the different array elements are not the same, so it should be expressed by an N-element vector $\rho ={\left[\begin{array}{ccc} \rho_{1}\cdots & \rho_{n}\cdots & \rho_{N} \end{array}\right]}^{T}$. An inconsistency between the estimated value of ρ and the actual value is undesirable. This will cause the estimator to be biased or even invalid.

### 3.3. Robust Estimator Based on Range Super Resolution

#### 3.3.1. Range Compression Based on Super Resolution

The multi-frequency maximum likelihood algorithm only accumulates the maximum likelihood function of each frequency point. However, expansion of the system bandwidth will effectively enhance the range resolution. Relying on this improvement, it is possible to distinguish the direct wave and reflected wave from the distance dimension. In order to further improve the range resolution, we adopted the super-resolution algorithm which can maintain the amplitude and the phase. Combining the array phase and the signal structure relationship of the direct and reflected paths, a low altitude angle estimation algorithm is proposed based on range super-resolution.

By separating the phase portions changed with frequency from those fixed in each element, the signal in Equation (7) in the *n*th element could be rewritten as follows

(10) $$s_{n}=A\varphi_{Dn}\left(f_{c},\theta \right)\cdot \phi_{Dn}\left(f_{B},\theta \right)+A\rho_{n}\varphi_{Rn}\left(f_{c},\theta \right)\cdot \phi_{Rn}\left(f_{B},\theta \right)+n_{n}$$

where

$$\varphi_{Dn}\left(f_{c},\theta \right)=exp\left\{-j2\pi \left(R_{D1}-ndsin\theta \right)\frac{f_{c}}{C}\right\}$$

$$\phi_{Dn}\left(f_{B},\theta \right)=exp\left\{-j2\pi \left(R_{D1}-ndsin\theta \right)\frac{f_{B}}{C}\right\}$$

$$\varphi_{Rn}\left(f_{c},\theta \right)=exp\left\{-j2\pi \left(R_{D1}+ndsin\theta_{R}+2H_{R}sin\theta \right)\frac{f_{c}}{C}\right\}$$

$$\phi_{Rn}\left(f_{B},\theta \right)=exp\left\{-j2\pi \left(R_{D1}+ndsin\theta_{R}+2H_{R}sin\theta \right)\frac{f_{B}}{C}\right\}$$

In Equation (10), *f_c_* is the center of the bandwidth while $f_{B}=f-f_{c}$. $\varphi_{Dn}\left(f_{c},\theta \right)$ and $\varphi_{Rn}\left(f_{c},\theta \right)$ only change with the array position, and they are the fixed phase portion in each element. Meanwhile, $\phi_{Dn}\left(f_{B},\theta \right)$ and $\phi_{Rn}\left(f_{B},\theta \right)$ vary when the frequency points are altered, and its bandwidth can guarantee the range information with the corresponding accuracy.

The Fourier transform is taken as an example to illustrate the range compression on each element, due to the uniform distribution of the frequency points. We use the compressed signal expression

(11) $$\begin{array}{l} s_{ℱn}=ℱ\left\{s_{n}\right\}\\ =A\varphi_{Dn}\left(f_{c},\theta \right)\cdot Bsinc\left\{\frac{B}{C}\cdot \left[r-\left(R_{D1}-ndsin\theta \right)\right]\right\}\\ +A\rho_{n}\varphi_{Rn}\left(f_{c},\theta \right)\cdot Bsinc\left\{\frac{B}{C}\cdot \left[r-\left(R_{D1}+ndsin\theta_{R}+2H_{R}sin\theta \right)\right]\right\}+n_{ℱn} \end{array}$$

in which sinc() is the Fourier transform of the rectangular window in the frequency domain. It represents the envelope of the pulse compression. After range pulse compression, the two sinc functions in $s_{ℱn}$ are the range envelopes of the direct wave and the reflected wave. They include the range migration which varies with the elevation angle and the element position. The remainder is the gain and the phase in the angular dimension independent of the range compression. It can be noticed that the phase difference between the array elements is only determined by the center frequency point *f_c_*, which no longer corresponds to a wide frequency band.

**Figure 4:**
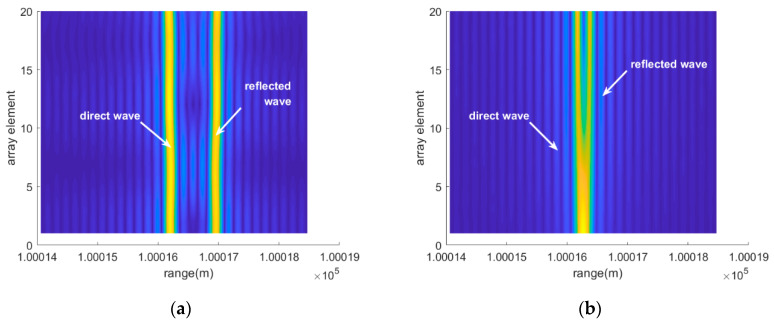
The RA graphs with FFT when the elevation angles are 6° and 1°, (**a**) and (**b**), respectively.

Figure 4 demonstrates the magnitude of the range compression results based on the data received by all of the array elements by performing fast Fourier transform (FFT). In Figure 4a, when the elevation angle is 6°, the direct and the reflected waves can be easily distinguished from the range-array (RA) surface due to the relatively large difference between their ranges. At the same time, it can be observed that the range migrates between the different array elements. So, the two lines formed by the range peaks are inclined along the array element dimension, with opposite tilt angles. That is also consistent with the wave propagation difference between the array elements. With increasing element position, the direct wave range gets shorter while the reflected range turns longer. However, when the elevation angle decreases to 1°, the range peaks of the lower elements overlap, making it difficult to resolve the two signals based on the range dimension. Simultaneously, the phases of the two range main lobes also interfere with each other at the overlapping position.

Therefore, in order to increase the angular resolution and extend the angular estimation lower limit, the range resolution need to be further improved. Considering the fixed system bandwidth, the super-resolution algorithm is adopted to complete the range compression. Considering the utilization of phase information along the array elements for the elevation angle estimation with the range information, the RELAX super-resolution algorithm is selected for its preservation of amplitude and phase.

The RELAX algorithm is a nonlinear least-squares relaxation algorithm, which can solve the parameter of the scattering model with fixed points. In the low elevation scenario, having only one target within the range sampling gate, the number of the scattering points is set to 2, and only the direct wave and the specular reflection wave will be recovered. The algorithm can also filter the interferences from the other scattering paths. The idea of the program CLEAN, in particular its iterative updating method, was introduced to solve the signal parameters [30].

The signal received by the *n*th array element is taken as an example to illustrate the RELAX algorithm

(12) $$s_{n}=\Omega a+e_{n}$$

in which

$$\Omega =\left[\varpi \left(r_{1}\right),\varpi \left(r_{2}\right),\cdots ,\varpi \left(r_{K}\right)\right],$$

$$\varpi \left(r_{k}\right)=\left[exp\left\{j2\pi r_{k}f_{1}\right\},exp\left\{j2\pi r_{k}f_{2}\right\},\cdots ,exp\left\{j2\pi r_{k}f_{M}\right\}\right],$$

$$a=\left[a_{1},a_{2},\cdots ,a_{K}\right],$$

and ***e****_n_* is the relaxed noise component.

The estimation of $\left\{{\hat{r}}_{k1},{\hat{r}}_{k2},{\hat{a}}_{k1},{\hat{a}}_{k2}\right\}$ can be obtained by minimizing the nonlinear least-squares (NLS) expression

(13) $$\left\{{\hat{r}}_{k1},{\hat{r}}_{k2},{\hat{a}}_{k1},{\hat{a}}_{k2}\right\}=\underset{\left\{\hat{r_{k1}},\hat{r_{k2}},\hat{a_{k1}},\hat{a_{k2}}\right\}}{\mathrm{argmin}}\|s_{n}-\Omega a{\|}_{2}.$$

The generalized inverse of ***Ω*** is defined as $\Omega^{+}={\left(\Omega^{H}\Omega \right)}^{-1}\Omega^{H}$.

${\hat{r}}_{k1}$ can be determined using the expression

(14) $$\left\{{\hat{r}}_{k1}^{1}\right\}=\underset{\left\{{\hat{r}}_{k1}\right\}}{\mathrm{argmin}}\|s_{n}-\Omega \Omega^{+}s_{n}{\|}_{2}.$$

Then, ${\hat{a}}_{k1}^{1}$ can be calculated by

(15) $$a=\Omega^{+}s_{n}$$

Removing the estimated signal $\left\{{\hat{r}}_{k1}^{1},{\hat{a}}_{k1}^{1}\right\}$ from the original signal $s_{n}$ yields

(16) $$s_{nk2}^{1}=s_{n}-{\hat{a}}_{k1}^{1}\varpi \left({\hat{r}}_{k1}^{1}\right)$$

The first estimation of the second signal can be obtained according to the following formula

(17) $$\left\{{\hat{r}}_{k2}^{1}\right\}=\underset{\left\{{\hat{r}}_{k2}\right\}}{\mathrm{argmin}}\|\left[I-\frac{\varpi \left({\hat{r}}_{k1}^{1}\right)\varpi^{H}\left({\hat{r}}_{k1}^{1}\right)}{M}\right]s_{nk2}^{1}{\|}_{2},$$

and

(18) $${\hat{a}}_{k2}^{1}=\frac{\varpi^{H}\left({\hat{r}}_{k1}^{1}\right)}{M}s_{nk2}^{1}.$$

The parameters of both signals can be updated alternately by iterating the previous two steps. When $\|s_{nk2}^{g+1}-s_{nk2}^{g}{\|}_{2}/\|s_{nk2}^{g}{\|}_{2}\leq \varepsilon$, the iteration can be stopped. The RELAX solution $\left\{{\hat{r}}_{k1}^{g},{\hat{a}}_{k1}^{g},{\hat{r}}_{k2}^{g},{\hat{a}}_{k2}^{g}\right\}$ can be outputted for further estimation. ${\hat{r}}_{k2}^{g}$ can be obtained by locating the dominant peak of the $\|\varpi^{H}\left({\hat{r}}_{k1}^{g}\right)s_{nk2}^{g}{\|}_{2}$. FFT can be used to implement the matrix multiplication which occupies the major calculation in the iteration. The number of FFT points is *MN_I_*, where *N_I_* is the multiple of the zero paddings. It is generally assumed that when the resolution of *MN_I_* points FFT can reach twice the expected accuracy, the reconstruction accuracy of RELAX can meet the requirements without introducing an excessive calculation burden.

**Figure 5:**
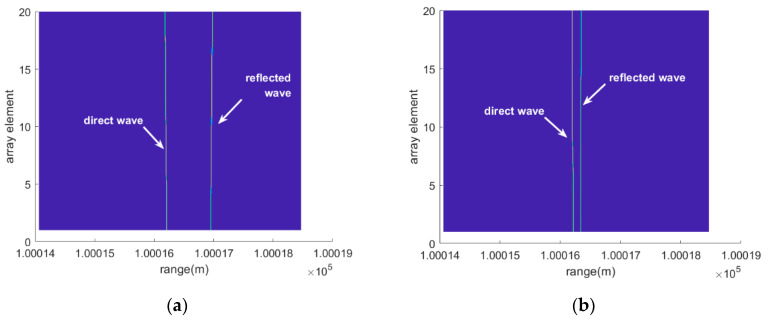
The RA graphs RELAX when the elevation angles are 6° and 1° in (**a**) and (**b**), respectively.

It can be clearly seen from Figure 5 that use of the super-resolution algorithms achieves a successful separation of the two waves on the RA surface even for a low angle of 1°. Since RELAX outputs sparse results, there is no problem with the side lobes interference. RELAX shows a better performance by utilizing the two targets prior assumption.

#### 3.3.2. The Robust Estimator

Range compression provides a signal-to-noise (SNR) ratio gain for the range peak position. However, due to the broadband effect, the range peaks of different array elements shift according to the elevation angle. Therefore, the signals of the two columns on the RA surface cannot be directly used to extract $\varphi_{Dn}\left(f_{c},\theta \right)$ and $\varphi_{Rn}\left(f_{c},\theta \right)$. A calibration for the range migration is essential.

The calibration matrix 𝓒(θ) is constructed as follow

(19) $$𝓒(\theta ){=}^{j2\pi \frac{dsin\left(\theta \right)f_{B}}{C}}.$$

The complex exponential terms contained in matrix 𝓒(θ) compensate for the range offset caused by the distance between the array elements being dependent on the elevation angle. The calibration essentially involved focusing the broadband frequency points on the center frequency. Since the angle of the direct wave is opposite to that of the reflected wave, 𝓒(θ) and its conjugate matrix are used to complete the range calibrations for the direct wave and reflected wave, respectively

(20) $$S_{𝒞D}\left(\theta \right)=S\circ 𝓒\left(\theta \right)$$

(21) $$S_{𝒞R}\left(\theta \right)=S\circ 𝓒{\left(\theta \right)}^{*}.$$

In these equations for range calibrations, ∘ presents the Hadamard product. The *n*th row vectors in $S_{𝒞D}\left(\theta \right)$ and $S_{𝒞R}\left(\theta \right)$ could be expressed as

(22) $$s_{𝒞Dn}\left(\theta \right)=A\varphi_{Dn}\left(f_{c},\theta \right)\cdot \phi_{𝒞DDn}\left(f_{B},\theta \right)+A\rho_{n}\varphi_{Rn}\left(f_{c},\theta \right)\cdot \phi_{𝒞DRn}\left(f_{B},\theta \right)+n_{𝒞Dn}$$

and

(23) $$s_{𝒞Rn}\left(\theta \right)=A\varphi_{Dn}\left(f_{c},\theta \right)\cdot \phi_{𝒞RDn}\left(f_{B},\theta \right)+A\rho_{n}\varphi_{Rn}\left(f_{c},\theta \right)\cdot \phi_{𝒞RRn}\left(f_{B},\theta \right)+n_{𝒞Rn}$$

where

$$\phi_{𝒞DDn}\left(f_{B},\theta \right)=exp\left\{-j2\pi \frac{f_{B}}{C}\left(R_{D1}\right)\right\},$$

$$\phi_{𝒞DRn}\left(f_{B},\theta \right)=exp\left\{-j2\pi \frac{f_{B}}{C}\left(R_{D1}-2ndsin\theta \right)\right\},$$

$$\phi_{𝒞RDn}\left(f_{B},\theta \right)=exp\left\{-j2\pi \frac{f_{B}}{C}\left(R_{D1}+2H_{R}sin\theta +2ndsin\theta \right)\right\},$$

$$\phi_{𝒞RRn}\left(f_{B},\theta \right)=exp\left\{-j2\pi \frac{f_{B}}{C}\left(R_{D1}+2H_{R}sin\theta \right)\right\},$$

and $n_{𝒞Dn}$ and $n_{𝒞Rn}$ are the noise vectors after calibration.

As one of the two calibration results, $S_{𝒞D}\left(\theta \right)$ achieves a decoupling of the angle and range information for the direct wave when the angle used for 𝓒(θ) is the same as the target angle. The calibration will not change the relationship between the ranges of the two waves. Correction of the direct wave range migration leads to the slope of the reflected wave being two times the slope of the original reflected wave. Despite this, in the range compressed RA signal, the two waves are separated in distance, and hence their interaction can be ignored. The angle and the range for the direct wave could be determined and investigated separately, a process also applicable to the reflected signal in $S_{𝒞R}\left(\theta \right)$.

$RA_{𝒞D}\left(\theta \right)$ and $RA_{𝒞R}\left(\theta \right)$ denote the output matrices of the super-resolution algorithms operating on $S_{𝒞D}\left(\theta \right)$ and $S_{𝒞R}\left(\theta \right)$, with $ra_{𝒞Dr}\left(\theta \right)$ and $ra_{𝒞Rr}\left(\theta \right)$ denote the column vectors these two matrices. *N_I_* is the multiple of the range interpolation

(24) $$RA_{𝒞D}\left(\theta \right)=\left[ra_{𝒞D1}\left(\theta \right),ra_{𝒞D2}\left(\theta \right)\cdots ,ra_{𝒞Dr}\left(\theta \right),\cdots ra_{𝒞DMN_{I}}\left(\theta \right)\right],$$

(25) $$RA_{𝒞R}\left(\theta \right)=\left[ra_{𝒞R1}\left(\theta \right),ra_{𝒞R2}\left(\theta \right)\cdots ,ra_{𝒞Rr}\left(\theta \right),\cdots ra_{𝒞RMN_{I}}\left(\theta \right)\right].$$

The peak positions of the different array elements of the direct wave in $RA_{𝒞D}\left(\theta \right)$ are aligned in range. The phases of each element can be directly extracted for angle estimation. Similarly, the phases of the reflected wave are also available in $RA_{𝒞R}\left(\theta \right)$.

To effectively integrate the angle information and the range relationship structure of the echo signal, we proposed an estimator designed to combines a maximum likelihood estimation of the array angle and a range peak search. After obtaining the direct wave range according to the first range peak, the elevation angle can be determined by finding the maximum value of Formula (26) in the angle search

(26) $$\hat{\theta }=\arg\underset{\theta }{\max}\left\{\frac{\|a^{H}\left(\theta \right)ra_{𝒞Dr}{\left(\theta \right)\|}^{2}}{\|a{\left(\theta \right)\|}^{2}}+\frac{\|a^{T}\left(\theta \right)ra_{𝒞R\left[r+\Delta r\left(\theta \right)\right]}{\left(\theta \right)\|}^{2}}{\|a^{*}{\left(\theta \right)\|}^{2}}\right\}.$$

where Δ*r*(*θ*) represents the range step-index according to the analysis of the range relationship between the two waves in Section 2. It is an integer calculated by rounding the quotient of the range difference in Equation (4) and the range resolution

(27) $$\Delta r\left(\theta \right)=⎣\frac{2H_{R}BN_{I}sin\theta }{C}+0.5⎦$$

The proposed estimator mimics an ML estimator, assuming that the $n_{𝒞Dn}$ and $n_{𝒞Rn}$ are still zero-mean Gaussian distribution after range compression by the super-resolution algorithm. Since RELAX is not a linear operation, the noises in the RA signal are not statistically independent of each other or have the same unknown covariance matrix. So, the proposed algorithm is an ML estimator only in an approximate sense. Due to the gain from the range compression, the effect of the noise at the range peak will also decrease proportionally. The estimator in Equation (26) assumes that the parameter related to the reflection coefficient is invariable in the reflection area, which indicates all the elements in ρ are equal. So, this estimator takes advantage of the phase of the reflected wave for angle estimation.

In practical applications, the distribution area of reflection points slides along the direction of the motion of the target. For low elevation angles, the area would be relatively large. Changes in the roughness, coverage and the grazing angle all lead to changes of the reflection coefficients both in amplitude and phase, and that coefficients corresponding to the different array elements will differ from each other and change constantly. As discussed above, for ML estimation, inaccurate reflection coefficients will cause model mismatch and estimation error. Therefore, when facing a non-ideal reflecting surface, without the prior information of the reflection coefficients, the amplitude-phase relationship of the reflected wave will provide no additional information for the angle estimation. Due to the direct waves propagating only through free space, the amplitude-phase relationship is relatively stable and reliable. As the range compression realizes the energy separation between the direct wave and the reflected wave, the distance structure between the two waves can provide robust but slightly less accurate angle information, according to its high wideband gain. Therefore, only the array phase of the direct wave is used with and the range structure to realize the angle estimation. Better robustness can be achieved with little gain loss. There is no need to estimate the reflection coefficients of different array elements when using this algorithm

(28) $$\hat{\theta }=\arg\underset{\theta }{\max}\left\{\frac{\|a^{H}\left(\theta \right)ra_{𝓒D\_SRr}{\left(\theta \right)\|}^{2}}{\|a{\left(\theta \right)\|}^{2}}+\|ra_{𝓒R\_SR\left[r+\Delta r\left(\theta \right)\right]}{\left(\theta \right)\|}^{2}\right\}.$$

## 4. Performance Analysis

The algorithm proposed in the current work achieves the angle estimation by employing high-precision range structure information. Therefore, the resolution and accuracy of the range estimation have a decisive impact on the angle estimation result. This section describes our analysis of system parameter requirements and the adaptability to the environment.

### 4.1. Lower Limit of the Angle Estimation

**Figure 6:**
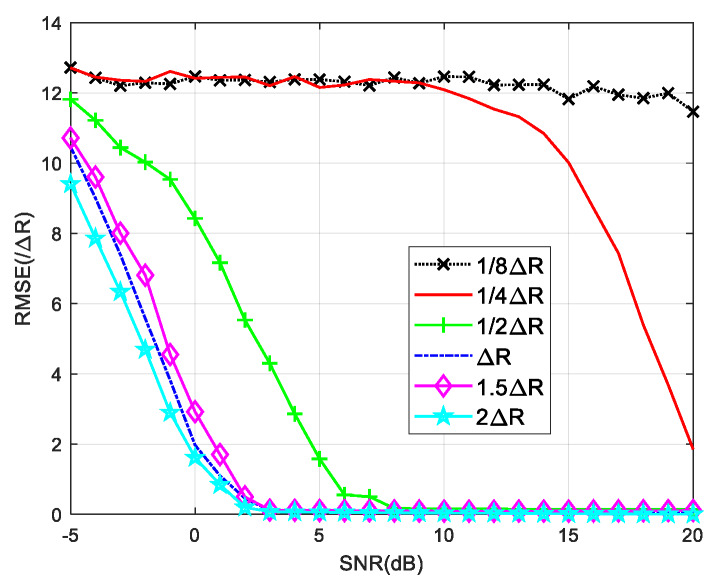
RMSE against input SNR with different range intervals by RELAX.

The range interval between the two signals affects the performance of the RELAX algorithm. Δ*R* is used to represent the resolution of the actual signal based on *B*. Figure 6 shows the root mean square errors (RMSEs) versus signal-to-noise ratio (SNR) calculated using RELAX of two signals separated from each other by various range intervals.

When the range interval between the two signals is greater than Δ*R*, the RMSE curves are found to be relatively stable with increasing range gap. A reliable estimation can be got for SNR values greater than 5 dB. When the range gap is between Δ*R*/2 and Δ*R*, the RMSE gradually increases as the range interval becomes narrow. The SNR required increases to 10 dB. When the two signals are close to each other in the range domain, i.e., with range intervals smaller than Δ*R*/2, the estimation fails. Δ*R*/2 is therefore chosen as the lower limit of the range interval between the signals, and is also used to determine the lower bound of the angle estimation based on the range super-resolution algorithm. We are able to calculate the required bandwidth based on the lower frequency limit to be measured

(29) $${\hat{\theta }}_{min}\approx arcsin\frac{C}{4B\left(H_{R}-Nd\right)}$$

### 4.2. Range Resolution Equivalent to Angular Resolution

The range variation corresponding to angular resolution Δ*θ* is calculated

(30) $$\Delta r=2H_{R}sin\theta -2H_{R}sin\left(\theta -\Delta \theta \right)\approx 2H_{R}sin\Delta \theta .$$

The number of the RELAX interpolation points required in one-way beacon mode can be calculated as

(31) $$N_{I}\geq \frac{C}{2H_{R}Bsin\Delta \theta }.$$

In the radar model including transceivers, the product of the bandwidth and the number of interpolation points need only be half that for the beacon mode.

The number of frequency points in the bandwidth determines the unambiguous distance range. Considering the distance migration of the direct and reflected waves, the number of frequency points M should be at least greater than that indicated by the equation:

(32) $$M\geq \frac{2B\left(H_{R}+Nd\right)sin\theta_{max}}{C}.$$

### 4.3. Surface Environment Adaptability

The variation in the ground cover in general affects the amplitude and phase of the reflection coefficient. At the same time, the surface fluctuations affect the distance of the reflected wave. According to Equation (28), it can be concluded that the phase changes of the reflection coefficients between different array elements do not affect the angle estimation. Attenuations in the amplitudes of the reflection coefficients will cause a proportional change in the SNR of the reflected wave, and hence decrease the estimation accuracy. The algorithm principle described in this paper is based on the combination of distance structure information and angle information. When the range variation caused by the height fluctuations in the reflection area becomes greater than the range resolution unit, the distance structure will change, directly leading to errors in the angle estimation. The height fluctuation of the reflecting surface needs to meet the requirement

(33) $$\Delta H_{RS}\leq \frac{C}{2BN_{I}sin\theta }.$$

Therefore, the use of a relatively flat terrain is recommended, or a known terrain elevation can be used as a priori information for compensation.

### 4.4. Requirements for Radar Systems

The algorithm proposed in this paper is based on a mono-pulse radar system with phased array. Generally, the higher carrier frequencies are easier to carry wideband signals, so it has a stronger capacity of low angle estimation when use the algorithm we proposed. As this algorithm integrates multiple frequency information within the whole frequency band, the integration process requires fast frequency agility of the radar system. For high-speed moving targets, a faster scanning time is essential to ensure that the range change caused by the target motion during the scanning time will not cross the distance resolution unit. Meanwhile, it is also necessary to increase the transmission power appropriately to compensate for the short signal duration for a sufficient received SNR.

## 5. Simulation

In this section, numerical simulations are executed to evaluate the proposed algorithm, demonstrating an improved robust performance in low elevation angle estimation.

We set simulation parameters for a typical wideband radar system in X band. A beacon model is applied in the simulation settings. All variables of the target state, the radar and the reflection area are parameterized. Different frequency points within the bandwidth are completely coherent.

The simulation parameters are listed in Table 1.

Multi-frequency RML and RSS algorithms are applied here as comparisons. Both of them can complete the angle estimation from the wideband signal. In RML, the reflection coefficient used to build the model is a fixed value −0.95. In RSS, based on three points near the estimated value of each angle, a total of six points were selected as the estimated angles to construct the focus matrix.

### 5.1. Robustness of the Reflection Coefficient

First, we verify the robustness of the algorithms to the reflection coefficient *ρ* through simulations. We illustrate the global performance of the four algorithms in terms of the RMSE versus the SNR and the elevation angle *θ* in three cases. In the first and second cases, the reflection coefficient is −0.95 and 0.7, respectively. In the third case, the amplitude and phase of the reflection coefficient have random distribution errors within a certain range.

**Figure 7:**
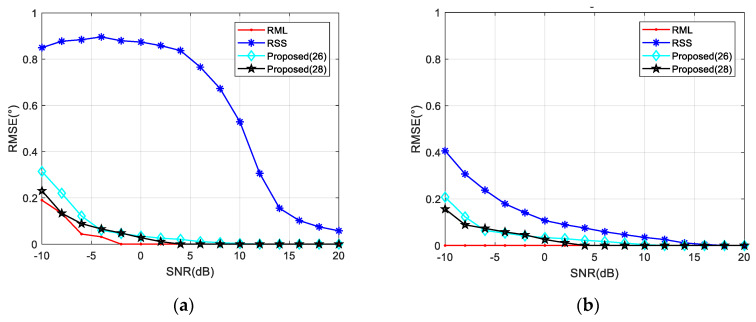
RMSE against input SNR in different elevation angles when *ρ* = −0.95. (**a**) *θ* = 1° and (**b**) *θ* = 5°.

**Figure 8:**
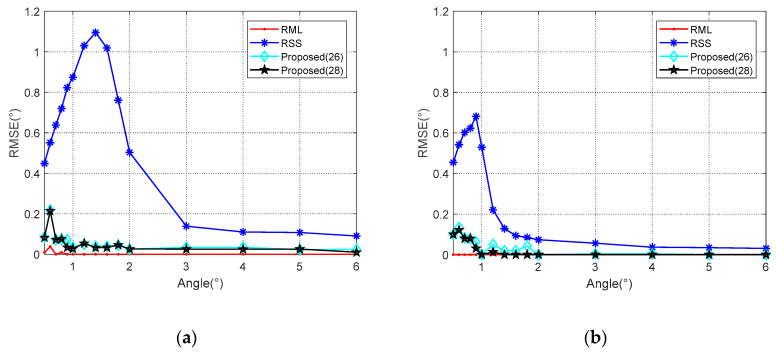
RMSE against elevation angle *θ* in different SNR when *ρ* = −0.95. (**a**) *SNR* = 0 dB; (**b**) *SNR* = 10 dB.

In the first case, when the signal parameters are completely consistent with the model parameters, the RML algorithm yields the smallest estimation errors for different SNR and *θ* valuse. When the elevation angle is reduced to 1°, RML only needs the SNR of about −7 dB to complete the angle estimation, and shows an RMSE of less than 0.1°. For SNRs less than 0 dB, an accurate estimation can be achieved along the angle interval of 0.5–6°. Unlike other algorithms, RSS analyzes reflected and direct waves as two targets. In comparison, RSS is less capable of measuring closely targets. As shown in Figure 7, the RSS yielded a significantly higher RMSE for an angle of 1° than for that of 5°. From Figure 8, it could be concluded that when the SNR is 0 dB, RSS will not be able to distinguish between the direct wave and the reflected signal with an angle below 1.4°. At an SNR of 10 dB, this limit is 0.9°. Compared with the proposed Algorithms (26) and (28), the noise performance of Algorithm (28) is slightly better than the other one, and it is more obvious when the SNR is low. That is because the range estimation brings higher robustness. The (28) algorithm can obtain the RMSE accuracy which is less than 0.1° when the SNR is greater than −6 dB. Such angle estimation accuracy equivalent to RML can be achieved if SNR is higher than 8 dB, consistent with the analysis in Section 4.1. Generally, the low elevation angle interval is defined as less than six degrees. With a sufficient SNR, Algorithm (28) can achieve accurate angle estimations for targets with elevation angles between 0.7 and 6°.

**Figure 9:**
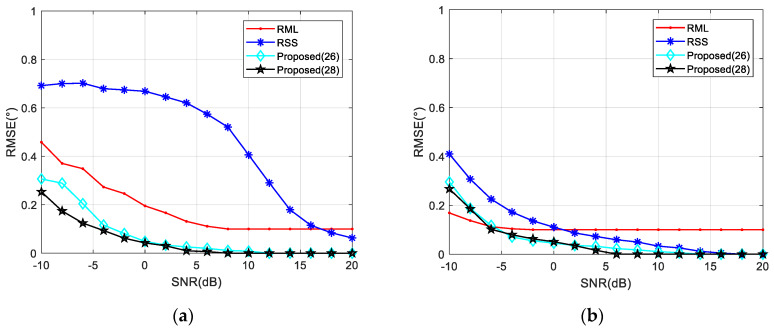
RMSE against input SNR in different elevation angles when *ρ* = 0.7. (**a**) *θ* = 1° and (**b**) *θ* = 5°.

**Figure 10:**
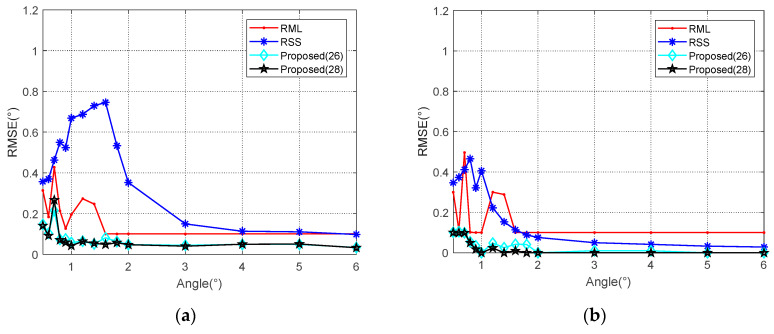
RMSE against elevation angle *θ* in different SNR when *ρ* = 0.7. (**a**) SNR = 0 dB; (**b**) SNR = 10 dB.

In the second case, both the amplitude and the phase of the reflection coefficient have fix error with the parameter *ρ* in the RML model. The simulation results are shown in Figure 9 and Figure 10. RML is obviously deteriorated with an angular deviation, even for sufficiently high SNR. The calculation process of RSS does not utilize the reflection coefficient. In this case, the overall performance of RSS improves due to the reduced energy of the reflected wave located on the negative angle axis. As the reflected energy declines, its interference to the direct wave also decreased accordingly. Despite this, the low angle estimation performance of RSS is still non-ideal. Similarly, with a reduced reflected wave energy, the noise performance of the Algorithm (28) would be expected to slightly decline. Nevertheless, it performs better than several other algorithms.

In the third case, both the amplitude and the phase of the reflection coefficient have random errors between each array element. Three different degrees of random distribution of *ρ* are listed in Table 2.

Among them, in *ρ*:case i, the difference of the *ρ* distribution between the array elements is the largest, while for *ρ*: case iii, it is the smallest.

**Figure 11:**
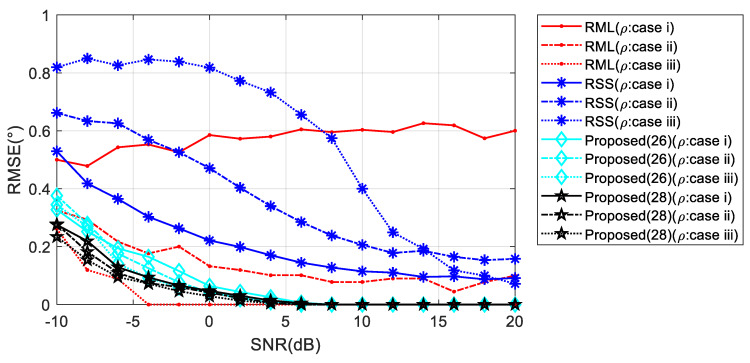
RMSE against input SNR with floating reflection coefficient when *θ* = 1°.

For low elevation angle, the phase difference between the array elements is small, and thus random distribution of *c* has a greater effect on the phases of the reflected wave. It can be observed in Figure 11, for *θ* = 1°, as the variation range of *ρ* rises, the RML and RSS performance decreases significantly. Meanwhile, Algorithm (28) exhibits stable performance.

**Figure 12:**
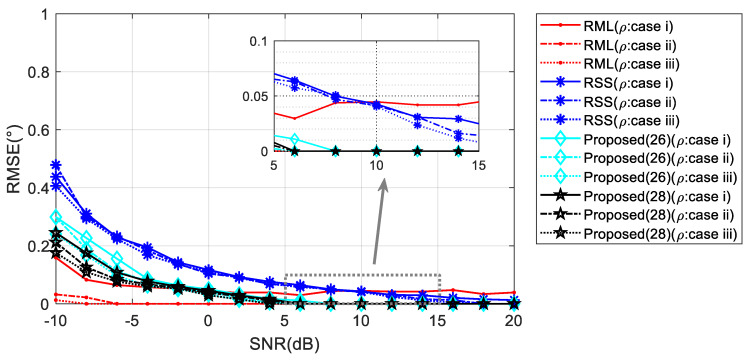
RMSE against input SNR with floating reflection coefficient when *θ* = 5°.

When the elevation angle gets higher, the influence of the reflection coefficient on the phase of the reflected wave is expected to decrease. The performances of the comparison algorithms improve correspondingly. As seen in the inset of Figure 12, RML performs best when the perturbation of *ρ* is small. However, in the presence of a large perturbation, the algorithm corresponding to Equation (28) could provide the highest angle estimation accuracy.

### 5.2. Robustness of Reflection Surface Height

Fluctuations in height on the reflecting surface were concluded, from the analysis in Section 4.3, to contribute errors to the estimated values produced by the proposed algorithm of the current work. Here, we also carry out simulation analysis of the presence of reflective surface height disturbances in the three cases. Table 3 lists three cases of height fluctuations from small to large.

**Figure 13:**
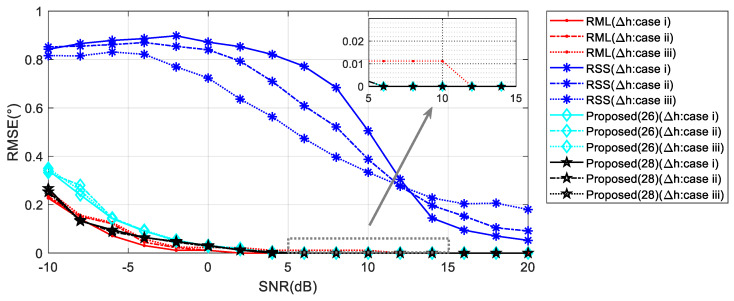
RMSE against input SNR with height fluctuation of reflecting surface when *θ* = 1°.

When the target elevation angle is relatively low, the perturbations on the range and element phases caused by the reflection surface height fluctuation are negligibly small. Therefore, in this case, the performances of the four algorithms does not change much. As shown in Figure 13, in the presence of large disturbances, however, the proposed Algorithm (28) proves superior to RML due to the combination of the phase of the direct wave, which is not affected by the surface height.

**Figure 14:**
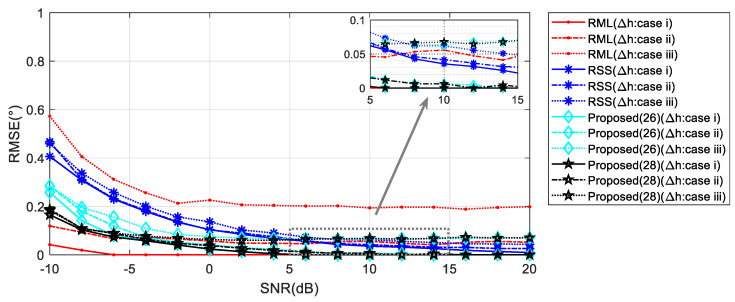
RMSE against input SNR with height fluctuation of reflecting surface when *θ* = 5°.

As the target elevation angle rises, the distance errors and phase errors increase proportionally in Figure 14. At this time, the RML has a poor performance when the disturbance is large. RSS has improved performance due to the increased angle. Algorithm (28) starts to appear in small errors depending on the level of the disturbance. When the reflection surface height fluctuation is 10 times the wavelength, the angle estimation accuracy is also less than 0.1°.

### 5.3. Bandwidth Performance

**Figure 15:**
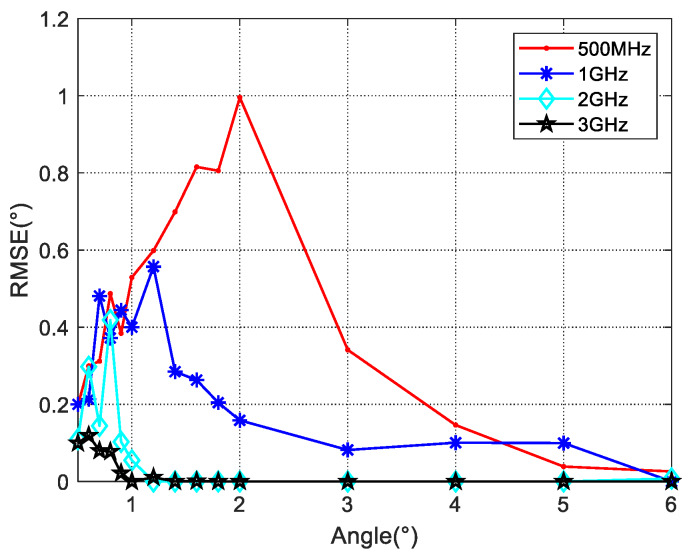
The performance of the estimator in Equation (28) with different values of bandwidth when *ρ* = −0.95 *SNR* = 10 dB.

The bandwidth determines the range resolution of the radar system. As analyzed in Section 4.1, the bandwidth determines the lower bound of the angle estimation based on the range super-resolution algorithm. The simulation of angle estimation capability under different bandwidth values is shown in Figure 15.

We compare the performance of the proposed Algorithm (28) with different bandwidth values. Assume that the SNR is 10 dB and the reflection coefficients are a constant value −0.95. As shown in Figure 15, to achieve the estimation angle RMSE less than 0.1°, the lower limit of the angle estimation is nearly 3° when the system bandwidth is 1 GHz, the angle lower limit rises above 4° when the bandwidth values decrease to 500 MHz. At a bandwidth of 2 Ghz, this limit is found to be around 1°. These results are consistent with the theoretical analysis in Section 4.1.

## 6. Conclusions

In this paper, a novel low elevation angle estimation scheme based on a wideband radar system is derived. The use of this scheme together with the RELAX super-resolution algorithm successfully separated the direct wave and reflected wave in the range dimension. The angle estimation could be obtained by synthesizing the phase characteristics between the array elements corresponding to the direct wave angle, and the range structure relationship between these two waves. We analyze the performance and robustness of the algorithm. Numerical simulations reveal the high precision of the proposed approach for a low elevation target. In addition, when compared with RML and RSS, the proposed algorithm demonstrates strong robustness to variations in the reflection coefficients and the reflecting surface height. In our further research, we plan to realize the low elevation angle estimation in a random frequency-hopping wideband system.

## Figures and Tables

**Table 1:** Simulation parameter.

**Radar parameters**	Bandwidth (*B*)/GHz	3
	Carrier frequency (*F_c_*)/GHz	9
	Radar height (*H_R_*)/m	4
	Number of Array Elements(*N*)	20
	Number of the frequency point(*Nf*)	32
	Interpolation points in RELAX	16

**Table 2:** Random distribution parameters of the reflection coefficient *ρ*.

Classification	Amplitude Distribution of *ρ*	Phase Distribution of *ρ*
*ρ*: case i	0.45 ~ 0.95	0 ~ 180°
*ρ*: case ii	0.65 ~ 0.95	80 ~ 180°
*ρ*: case iii	0.85 ~ 0.95	160 ~ 180°

**Table 3:** Random distribution of the reflection surface height fluctuation Δ*h*.

Classification	Height Distribution
Δ*h*: case i	0 ~ 1*λ* ^1^
Δ*h*: case ii	0 ~ 5*λ*
Δ*h*: case iii	0 ~ 10*λ*

^1^*λ* is the wavelength corresponding to *f_c_*

## Appendix A

Considering the *R_D_* can be directly acquired by range detection, we describe the multipath geometry relation in polar coordinates.

The range difference between the direct path and the reflected path is

(A1) $$\Delta R_{n}=R_{Rn}-R_{Dn}=R_{R1}-R_{D1}-\left(n-1\right)d\left(sin\theta_{R}-sin\theta_{D}\right).$$

Using the approximation in Equation (3), we have

(A2) $$\Delta R_{n}=R_{R1}-R_{D1}+2\left(n-1\right)dsin\theta_{D}.$$

According to the geometry relations

(A3) $$R_{R1}=\sqrt{{\left(R_{D1}cos\theta_{D}\right)}^{2}+{\left((R_{D1}sin\theta_{D}+2H_{R}\right)}^{2}}=\sqrt{R_{D1}^{2}+4H_{R}R_{D1}sin\theta_{D}+4H_{R}^{2}}.$$

Equation (A3) can be expanded by the binomial theorem. In the low elevation circumstance, except for the first two orders, the higher-order terms in the expansion can be neglected since they are too small. It follows that

(A4) $$R_{R1}\approx R_{D1}+\frac{4H_{R}R_{D1}sin\theta_{D}+4H_{R}^{2}}{2R_{D1}}\approx R_{D1}+2H_{R}sin\theta_{D}.$$

Hence,

(A5) $$\Delta R_{n}\approx 2sin\theta_{D}[H_{R}+\left(n-1\right)d].$$
